# Use of a new transanal irrigation device for bowel disorder management by patients familiar with the irrigation technique: a prospective, interventional, multicenter pilot study

**DOI:** 10.1007/s10151-020-02212-x

**Published:** 2020-04-21

**Authors:** K. Charvier, V. Bonniaud, D. Waz, C. Desprez, A.-M. Leroi

**Affiliations:** 1 Department of Physical Medicine and Rehabilitation, Henry Gabrielle Hospital, Lyon University Hospital Center, 69230 St Genis Laval, France; 2 Department of Physical Médicine and Rehabilitation, Le Bocage Hospital, Dijon Regional University Hospital Center, Dijon, France; 3 Department of Digestive Physiology and CIC-CRB 1404, Normandie Univ, UNIROUEN, INSERM U1073, Rouen University Hospital, Rouen, France

**Keywords:** Transanal irrigation, Balloon catheter, Safety, Efficacy, Electric pump

## Abstract

**Background:**

The aim of this study was to evaluate the feasibility of transanal irrigation (TAI) with a new medical device incorporating an electric pump, the IryPump®R Set.

**Methods:**

An interventional, prospective, open-label, non-comparative, multicenter pilot study on TAI was conducted at three French university hospitals. Patients with experience of TAI were enrolled for a 1-month period during which 5 consecutive TAIs were performed using the IryPump®R Set (B.Braun Melsungen AG Melsungen, Germany). The study’s primary efficacy criterion was successful TAI, defined as (i) use of the patient’s usual irrigation volume of water, (ii) stool evacuation, and (iii) the absence of leakage between TAIs. The first two TAIs were not taken into account in the main analysis. The secondary outcome measures were device acceptability, bowel dysfunction scores, tolerability, and safety.

**Results:**

Fifteen patients were included between November 2016 and May 2017, and 14 were assessed in the main analysis. The TAI success rate was 72.4% (21 out of 29 procedures). The bowel dysfunction scores at the end of the study did not differ significantly from those recorded on inclusion. A high proportion of patients (> 70%) reported that TAI was feasible with the new medical device. There were no serious adverse events or device-related adverse events. At the end of the study, 50% of the participants were willing to consider further use of the new device.

**Conclusions:**

In patients familiar with TAI, using a new medical device incorporating an electric pump was feasible. Levels of patient satisfaction were high, especially with regard to comfort of use and a feeling of security during TAI.

**Electronic supplementary material:**

The online version of this article (10.1007/s10151-020-02212-x) contains supplementary material, which is available to authorized users.

## Introduction

Bowel dysfunction (incontinence and/or constipation) is a major concern, since the (non)evacuation of feces cannot be consciously controlled [1, 2]. In some cases, conservative measures (such as diet, laxatives or antidiarrheals, digital stimulation) may provide the patient with sufficient relief [3, 4]. However, the rectum and distal portion of the sigmoid colon can be emptied by introducing water into the anus (typically through a balloon catheter or a cone catheter). This transanal irrigation (TAI) procedure can enable a patient to schedule the regular evacuation of stools, and thus enhance his/her activities of daily living [5–8]. For example, it has been reported that TAI over a 6-month period led to a significant improvement in quality of life for patients with low anterior resection syndrome [9]. Long-term TAI can be associated with good but variable results. In a questionnaire-based survey of 169 patients with impaired defecation and who did not respond to drug treatment and biofeedback therapy, the overall success rate was 45% [10]. However, the long-term continuation rate for TAI was low for patients with soiling or fecal incontinence and high for those with obstructed defecation and those who had had low anterior resection or pouch surgery [10]. Similarly, Vollebregt et al. found that long-term continuation was moderate (only 45% of patients continued for a median time of 12 months), but that persistence with TAI was associated with better scores on the SF-36 subscale energy/fatigue subscale [11]. In a study of 49 consecutive patients with multiple sclerosis, the continuation rate after a mean follow-up period of 40 months was 55% [12]. Bildstein et al. reported that only 43% of TAI-trained patients with constipation or fecal incontinence continued to use TAI 1 year after the training session [13].

Hence, long-term TAI may be suitable for some patients. However, the introduction of a catheter into the rectum is not always easy or risk-free [14]. First, good manual dexterity is required, and training is essential [15]. Second, there is a very low risk of potentially life-threatening perforation of the rectal wall by the end of the catheter or an over-inflated balloon catheter. Other side effects of TAI include abdominal cramping, dizziness, chills, nausea and/or minor rectal/anal bleeding [16]. Third, the presence of fluid in the rectum (typically between 500 ml and 1000 ml, in adults) can sometimes induce autonomic dysreflexia phenomena [14]. Overall, an individual’s wish and/or ability to initiate or continue to perform TAI is a multifaceted matter that involves physical factors (manual dexterity, core stability, ability to transfer to a commode), negative side effects (such as leakage, discomfort, bleeding) and psychological factors. Hence, patient education and nurse support appear to be essential for—but do not guarantee—persistence [15, 17].

Various medical devices for TAI (based on gravity-driven instillation, hand pumps or electric pumps) are on the market [6]. A well-known device (the only one currently reimbursed by the French national health insurance system) is the Peristeen® Anal Irrigation System (Coloplast A/S, Humlebaek, Denmark) [8, 18]. The aim of the present pilot study of patients familiar with TAI was to establish whether this procedure was feasible with a new, recently CE-marked pump system—the IryPump®R Set (B.Braun Melsungen AG Melsungen, Germany).

## Materials and methods

### The study population

Given the difficulty of evaluating a TAI system in patients who have never performed this procedure before, we decided to enroll experienced Peristeen® users for evaluation of the IryPump®R Set over a series of 5 consecutive TAIs. Patients referred to the rehabilitation departments of two French university hospitals and to the physiology unit of another from November 2016 to May 2017 were considered for inclusion if they had performed TAI satisfactorily (alone or with help) for at least 6 weeks. The other inclusion criteria were: age ≥ 18, informed consent, neurogenic or non-neurogenic bowel disorders, and constipation and/or fecal incontinence (defined according to the Cleveland Clinic Constipation Score (CCCS) [19], and the St. Mark’s Incontinence Score (SMIS), respectively [20]). The main exclusion criteria were pregnancy, participation in another clinical study, and the standard contraindications for TAI (intestinal obstruction, inflammatory bowel disease, ischemic colitis, colorectal carcinoma, anal or rectal prolapses or fistulas, anal or colorectal surgery or endoscopic polypectomy in the preceding 3 months, anal or colorectal stenosis, and acute diverticulitis).

The study’s objectives and procedures were approved by an independent ethics committee (*CPP Sud-Est III*, Bron, France) on June 21st, 2016, and the study was registered at www.clinicaltrials.gov (NCT02944916). The study protocol was performed in compliance with French and European legislation and the tenets of the Declaration of Helsinki. All included patients received information on the study’s objectives and procedures, and gave their written consent prior to participation.

### Study design

We performed an interventional, prospective, open-label, non-comparative, multicenter pilot study. The participants learned to use the new TAI system during a training session organized in an investigating center. Each participant was asked to perform a total of 5 consecutive TAIs with the study device during the following month (including the first TAI in hospital, under the investigator’s supervision, constituting the study inclusion visit). The 2nd to 5th TAIs were performed at the patient’s home and at the time of day of their choosing. During the 1 month evaluation period, the study participants could phone the investigating center if they had questions. After the evaluation period, the patient attended the hospital and the system was evaluated. The following data were recorded at the inclusion visit: demographic data, medical history, the usual pattern of TAI with the Peristeen® (volume of water, number of presses for balloon inflation, occurrence of leakage), the baseline bowel disorder scores (the Neurogenic Bowel Dysfunction Score (NBDS) [21], the CCCS [19], and the SMIS [20]). At each TAI during the 1-month evaluation period, the patient recorded the following information in a study diary: the date and time of the TAI, any leakage since the previous TAI, the volume of water instilled, the number of presses used for balloon inflation, the consistency of the fecal output (on the Bristol scale), the success of the procedure, the total time needed to perform the procedure, any occurrence of leakage during the procedure, and the use of accessories such as a protection pad or rectal tampon. At the end of the evaluation period, the patient filled out a questionnaire on the ease of preparation, ease of use, comfort of use, feeling of security, and overall satisfaction (all rated on a 1-to-4 Likert scale: 1 “not satisfied”, 2 “not very satisfied”, 3 “satisfied”, or 4 “very satisfied”), and willingness to use the device in the future (yes or no). At the end-of-study visit, the investigator recorded adverse events (AEs), if any the number of unused catheters returned, the number of catheters recorded in the patient diary, the NBDS, CCCS and the SMIS, and any changes in concomitant medications. The AEs were coded according to the Medical Dictionary for Regulatory Activities (www.meddra.org).

### The study device

The study device was the IryPump® R Set (B.Braun Melsungen AG, Melsungen, Germany). This set included several individually CE-marked class IIa medical devices: an electric pump within a station (the IryPump® R Station), a water container (IryPump® Container), a tube (IryTube®) and a rectal catheter (IryCath®) (Electronic Supplementary Material). Each included patient was supplied with the IryPump® R Set and an additional pack of 10 disposable rectal catheters. The patients were told that although the overall procedure for TAI was very similar to that for the Peristeen®, the IryPump® R Set had some important distinguishing features. First, the electronically controlled pump enables finer adjustment of the irrigation rate, relative to a manual pump. Second, the IryCath® balloon catheter has two interconnected, inflatable sections: the first locates in the rectal ampulla, and the second locates in the anal canal. This design is intended to minimize leakage during the TAI procedure.

### Primary criterion for evaluation

The study’s primary objective was to describe the IryPump®’s efficacy (i.e., the success of TAI). Successful TAI was defined as a two-stage composite endpoint. First, the study personnel judged whether the volume of water used in the TAI was similar to the average reported previously with the Peristeen®. Second, the 2nd to 5th TAIs were scored by the patient for (i) the successful evacuation of stools, and (ii) the absence of anal stool leakage before the next TAI (or, for the last TAI in the study, in the 48 h following irrigation); a successful TAI had to meet both of this conditions. To avoid bias related to unfamiliarity with the study device, we decided not to take account of the first two TAIs in the primary analysis. Hence, the primary outcome measure was considered for the 3rd, 4th and 5th TAIs only, referred to henceforth as “relevant TAIs”. Next, relevant TAIs meeting the composite endpoint were classified as “successful TAIs”.

### Secondary criteria for evaluation, including safety

The following secondary outcome measures were assessed: the device’s ease of preparation, handling and use (on 1-to-3 or 1-to-4 Likert scales) after the first TAI; the three validated bowel dysfunction scores (NBDS, CCCS, and SMIS) at the start and end of the evaluation period; comfort of use, the feeling of security, overall satisfaction, willingness to use the device in the future, and AEs.

### Sample size calculation and statistical analyses

Given the absence of literature data on the primary outcome measure, the target sample size (15 patients and 45 relevant TAIs) in this pilot study was based on the investigating centers’ expected recruitment capacity and the length of the recruitment period, rather than on a statistical calculation. Each investigating center was asked to recruit 5 patients. The full analysis set (FAS) comprised all patients in the safety population with at least one evaluation of the primary outcome criterion. Descriptive statistics were performed using SAS software (version 9.4, SAS Institute Inc., Cary, NC, USA). Quantitative variables were defined as the mean ± standard deviation or the median (range). Categorical variables were defined as the number (%). Baseline vs. end-of-study data were compared using Student’s *t *test or Wilcoxon’s signed-rank test, depending on the data distribution. The threshold for statistical significance was set at *p*<0.05.

## Results

### Characteristics of the study population

**Fig. 1:**
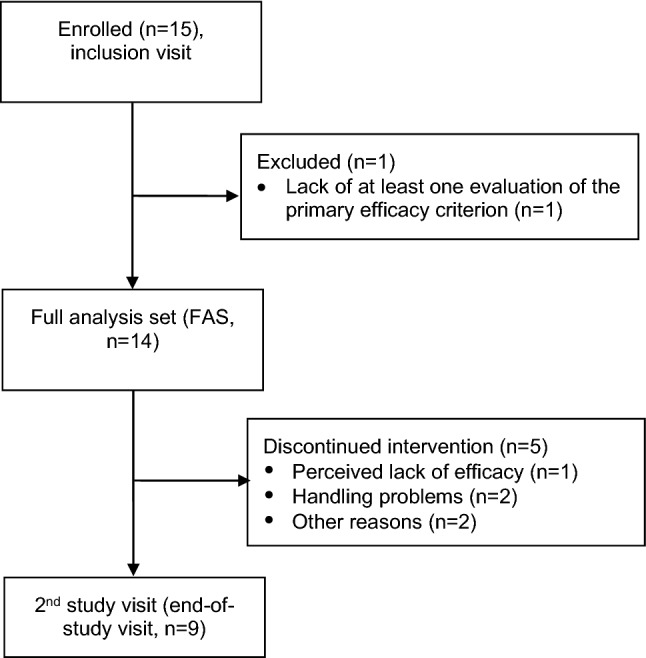
Study flowchart

A total of 15 patients (5 females, 8 males; median age: 53 years; age range: 35-76 years) were included in the study between November 2016, and May 2017 (Fig. 1). One of the included patients did not have data for the 2nd to 5th TAIs, and so the FAS comprised 14 patients.

The demographic and clinical characteristics of the FAS (including data on the patients’ prior use of the Peristeen® device) are summarized in Table 1. Other than oral laxatives, adjunct treatments were rare. It is noteworthy that at baseline, the severity of the bowel disorder (according to the NBDS) was not homogeneous; all but one of the patients had either a very severe disorder or, in contrast, a very mild disorder. All but 2 of the patients had moderate-to-severe constipation, according to the CCCS. Six of the patients had severe or very severe fecal incontinence, according to the SMIS. All the patients had been using the Peristeen® for much longer than the 6 weeks prespecified as an inclusion criterion: the length of use ranged from 1.17 to 7.50 years.

**Table 1:** Demographic and clinical characteristics of the study population (FAS, *n* = 14)

Characteristic	Study population (FAS, *n* = 14)
Sex, male, *n* (%)	8 (57.1%)
Age (years), mean ± SD, (range)	54.3 ± 12.1 (35–76)
Cause of bowel disorder, *n* (%)	
Neurologic^a^ Non-neurologic^b^	11 (78.6%) 3 (21.4%)
Fecal incontinence, *n* (%)	6 (42.9%)
Chronic constipation, *n* (%)	12 (85.7%)
Duration (years) in practicing TAI with the Peristeen®, mean ± SD [median], range	4.3 ± 2.3 [4.5], 1.17–7.50
Current volume of water used during the Peristeen® procedure (ml), mean ± SD [median]	775.0 ± 192.9 [800.0]
Current duration of the TAI procedure with the Peristeen®, (min), mean ± SD [median]	31.8 ± 14.0 [30.0]
Additional treatments/procedures, *n* (%)	
Diet Abdominal massage Oral laxatives Suppository Anal or peri-anal stimulation Antidiarrheals	1 (7.1%) 4 (28.6%) 7 (50.0%) 1 (7.1%) 2 (14.3%) 1 (7.1%)
Solid stool leakage between two TAIs over the previous 6 weeks	3 (21.4%)
Liquid stool leakage between two TAIs over the previous 6 weeks	9 (64.3%)
Satisfied or very satisfied with TAI	13 (92.9%)
NBDS^c^, mean ± SD [median]	10.09 ± 6.12 [6.00]
Severity of bowel dysfunction^c^, according to the NBDS	
Very minor Minor Moderate Severe	6 (54.5%) 1 (9.1%) 0 (0%) 4 (36.4%)
CCCS, mean ± SD [median]	8.36 ± 4.45 [7.00]
Severity of constipation, according to the CCCS	
Mild Moderate Severe	2 (14.3%) 7 (50.0%) 5 (35.7%)
SMIS, mean ± SD [median]	10.50 ± 6.10 [8.00]
Severity of fecal incontinence, according to the SMIS	
Mild Moderate Severe Very severe	1 (7.1%) 7 (50.0%) 5 (35.7%) 1 (7.1%)

*FAS* full analysis set, *SD* standard deviation, *TAI* transanal irrigation, *NBDS* Neurogenic Bowel Dysfunction Score, *CCCS* Cleveland Clinic Constipation Score, *SMIS* St. Mark’s Incontinence Score

^a^Paraplegia (*n* = 5), cauda equina syndrome (*n* = 3), paraparesis (*n* = 1), caudal regression syndrome (*n* = 1) and multiple sclerosis (*n* = 1)

^b^Rectal surgery (*n* = 2) and idiopathic constipation (*n* = 1)

^c^Data from the 11 participants with neurologic bowel dysfunction only

Five of the patients (35.7%) withdrew early from the study; in 4 of these cases, the patient withdrew before the 3rd TAI and thus did not contribute any relevant TAIs. Patient #0101 withdrew because the IryCath® was thought to be less lubricated than the Peristeen® catheter and because the pump’s battery took 5 hours to charge. Patient #0102 considered that he would not be able to use the new device at home. Patient #0105 experienced fatigue following the first TAI and subsequently abandoned TAI in general (i.e., including irrigations with the Peristeen®). Patient #0201 considered that the IryCath® was too large. Patient #205 experienced water leakage during 3 TAIs. Hence, 9 patients (64.3%) completed the study after a series of 5 TAIs. The mean length of follow-up was 20.2 ± 14.7 days.

### The primary outcome measure (efficacy)

A total of 56 TAIs were recorded by the 14 patients in the FAS. Stools were eliminated in 47 of the 56 TAIs (83.9%). The mean volume of water per irrigation was 762.5 ± 281.8 ml, and the mean duration of the procedure was 29.0 + 11.7 minutes. Water leakage occurred during 28 TAIs (50%). Twenty-nine of these 56 TAIs (performed by 10 of the 14 patients) were considered to be “relevant” (10 third TAIs, 10 fourth TAIs, and 9 fifth TAIs; Table 2) and were thus included in our analysis of the primary outcome. As mentioned above, 4 of the 14 patients did not have any relevant TAIs because they withdrew from the study before having performed 3 or more TAIs. Of the 29 relevant TAIs, 21 (72.4%) in 8 patients were judged to be successful. For 6 of these 8 patients, all 3 relevant TAIs were successful. For 1 patient, 2 relevant TAIs were successful. For the last patient, only 1 relevant TAI was successful. Hence, 2 of the 10 patients with relevant TAIs did not have any successful TAIs. When considering the 8 relevant TAIs that were not successful (Table 3), the main problem (in 7 TAIs) was leakage after the procedure, even though satisfactory output had been achieved. However, patients #0301 and #0304 reported that they had also experienced leakage between TAIs with the Peristeen®. Lack of satisfactory output was noted for 3 TAIs; this was primarily due to water leakage during the TAI itself.

**Table 2:** Characteristics of the relevant TAIs (*n* = 29) and those performed prior to the study with the Peristeen® by the corresponding 9 patients

Variable	Relevant TAIs with the IryPump® R (*n* = 9 patients)	Typical TAIs with the Peristeen® (*n* = 9 patients)
Water volume, ml	839.7 ± 181.0 [900]	805 ± 173.9 [800]*
Number of pump presses	3.9 ± 0.6 [4]	3.40 ± 0.70 [3.5]*
Duration of TAI, minutes	30.5 + 9.0 [30]	33.5 ± 14.5 [30]*

Data are quoted as the mean ± SD [median]

*SD* standard deviation, *TAI* transanal irrigation

*p > 0.05, non-significant

**Table 3:** Patients with at least one unsuccessful TAI

Patient #	Irrigation #	Satisfactory stools output?	Leakage after the TAI?	Successful TAI?
0104	3	No	No	No
0201	3	No	Yes	No
	4	No	Yes	No
0301	3	Yes	Yes	No
	4	Yes	Yes	No
0304	3	Yes	Yes	No
	4	Yes	Yes	No
	5	Yes	Yes	No

Ten patients had relevant TAIs. Of these, the 4 specified in this table had a mixture of successful and unsuccessful TAIs. For the other 6 patients, all relevant TAIs were successful

*TAI* transanal irrigation

### Secondary outcome measures

**Fig. 2:**
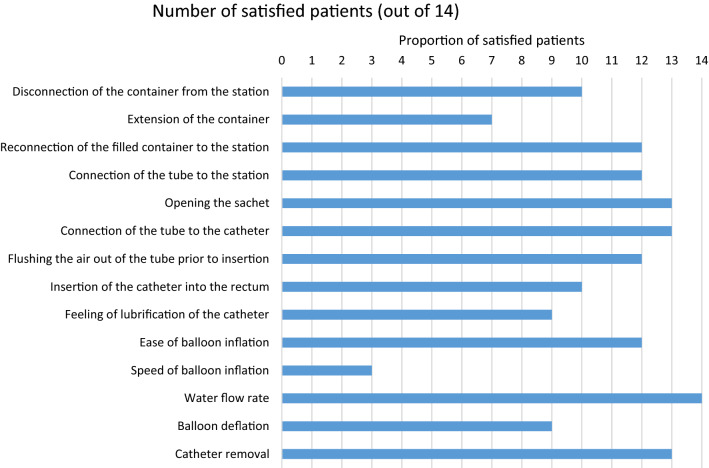
Satisfaction (“satisfied” or “very satisfied”) with a range of device features in the full analysis set (*n* = 14)

**Fig. 3:**
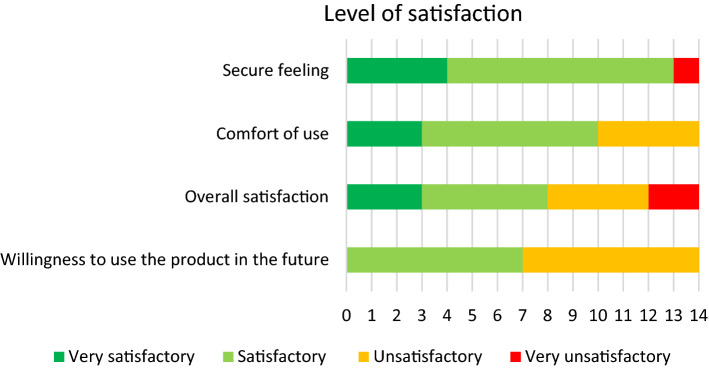
Levels of patient satisfaction at the end of the study

The bowel dysfunction scores rated at the end of the study did not differ significantly from those recorded on inclusion (Wilcoxon signed rank test: *p *= 0.25 for the NBDS, *p *= 1.00 for the CCCS, and *p* = 0.25 for the SCIS; Table 4). Water leakage occurred during 28 of the 56 TAIs (50%), and stools were eliminated in 47 procedures (83.9%). Despite their unfamiliarity with the new device, a high proportion of patients reported that the IryPump®R Set was very easy or easy to prepare and use for the first TAI (> 70% of the patients for most features, and even > 80% or > 90% for some features; Fig. 2). At the end of the study, levels of patient satisfaction were high (Fig. 3)—especially with regard to comfort of use and a feeling of security during TAI. The overall satisfaction rate was somewhat lower (57%), and 50% were willing to consider use of the IryPump®R Set in the future; we ascribe this to the participants’ familiarity and satisfaction with the Peristeen®.

**Table 4:** Bowel dysfunction scores at the end of the study, as given by the participants

Variable	Values at the start of the study	Values at the end of the study
NBDS, mean ± SD [median]	10.09 ± 6.12 [6.00] (*n* = 11 participants*)	12.50 ± 8.20 [10.50] (*n* = 10 participants*)
Severity of bowel dysfunction in participants with neurologic bowel dysfunction, according to the NBD score		
Very minor Minor Moderate Severe	6 (54.5%) 1 (9.1%) 0 (0%) 4 (36.4%)	4 (40.0%) 1 (10.0%) 1 (10.0%) 4 (40.0%)
CCCS, mean ± SD [median]	8.36 ± 4.45 [7.00] (*n* = 14 participants)	8.46 ± 4.86 [8.00] (*n* = 13 participants)
Severity of constipation, according to the CCCS		
Mild Moderate Severe	2 (14.3%) 7 (50.0%) 5 (35.7%)	2 (15.4%) 6 (46.2%) 5 (38.5%)
SMIS, mean ± SD [median]	10.50 ± 6.10 [8.00] (*n* = 14 participants)	9.92 ± 6.60 [10.00] (*n* = 13 participants)
Severity of fecal incontinence, according to the SMIS		
Mild Moderate Severe Very severe	1 (7.1%) 7 (50.0%) 5 (35.7%) 1 (7.1%)	3 (23.1%) 5 (38.5%) 5 (38.5%) 0

^*^Data for the participants with neurologic bowel dysfunction only

*SD* standard deviation, *TAI* transanal irrigation, *NBDS* Neurogenic Bowel Dysfunction Score, *CCCS* Cleveland Clinic Constipation Score, *SMIS* St. Mark’s Incontinence Score

### Tolerability and safety

A high proportion (92.2%) of the study participants (all long-term Peristeen® users) were satisfied or very satisfied with the IryPump®R Set. When considering the total of 56 TAIs, discomfort was reported in 8 procedures (14.3%) by 4 different patients. This discomfort was mild in 5 cases, moderate in 1 case, and severe in 2 cases (25.0%). The discomfort occurred solely during catheter insertion in 7 of the 8 instances, and during both catheter insertion and stool evacuation in the 8th instance. Pain was reported during 5 TAIs (mild pain in 4 cases and moderate pain in 1 case).

Of the 15 patients in the safety population, 4 (26.7%) experienced at least one AE. A total of 14 AEs were reported: abdominal pain (6 events in 2 patients); rectal bleeding (1 event in 1 patient), chills (5 events in 1 patient); a urinary tract infection (1 event in 1 patient), and bronchial pneumonia (1 event in 1 patient). A patient with a history of autonomic dysreflexia events during TAI with his usual device reported a total of 10 events: 5 episodes of chills of moderate intensity during TAI, 4 episodes of mild pain during TAI, and a urinary tract infection. The rectal bleeding event occurred in a patient with a history of hemorrhoids. There were no serious AEs. One of the abdominal pain events led to study discontinuation. None of the AEs were considered to be directly related to the device itself (e.g., device failure). All the events resolved without treatment, and there were no sequelae.

## Discussion

The results of this interventional, prospective, open-label, non-comparative, multicenter pilot study showed that the IryPump®R Set TAI device was associated with a success rate (normal water volume, satisfactory bowel evacuation, and no leakage between TAIs) of 72.4%. When considering all the TAIs performed (including those that did not correspond to the patient’s usual irrigation volume or time), stools were evacuated in 83.9% of the procedures. The frequency of leakage of the irrigation fluid around the balloon was relatively high (in 50% of procedures), although similar values have been reported in the literature [16]; this is supposedly a normal event, and may have been due to the patient’s lack of familiarity with the new device [22]. Use of the IryPump®R Set did not appear to influence bowel dysfunction scores, although the 1-month data collection period was short. Most participants found the device easy, comfortable, and safe to use. According to the questionnaire, one of the most popular device features was the use of electric pumps (rather than manual pumping) to inflate the balloon catheter and to instill water for TAI. However, 9 of the participants (64.3%) complained that the instillation rate was too low—although increasing this might conceivably raise safety concerns with regard to the potential triggering of autonomic nervous system disorders. Three of the participants (21.4%) stated that the upper part of the water container was very difficult to extend before filling. Despite being very satisfied with their usual TAI device (Peristeen®), half of the trial participants stated said they would be willing to use the IryPump®R Set device in the future. The occurrence of some technical problems (especially when starting to use a new TAI device) may not dissuade patients from long-term use. In a study of long-term use of the Peristeen® by 16 patients, the mean satisfaction score was 9.2 out of 10—even though over three-quarters of the long-term users had experienced technical problems at some point [23].

The purpose of the present pilot study was to assess the IryPump® R Set’s feasibility for TAI after basic training in the new device had been provided to experienced Peristeen® users (i.e., patients already familiar with TAI). This is why we chose to focus on “relevant” TAIs; we wanted to assess the device under conditions that might reflect regular use by a trained user. Hence, we did not take account of the first two TAIs performed by each patient. Interestingly, the technical results for all TAIs were similar to those for relevant TAIs only; the respective mean volumes of water per irrigation were 762.5 ± 281.8 ml and 839.7 ± 181.0, and the respective mean durations were 29.0 + 11.7 min and 30.5 + 9.0 min.

The proportion of the 56 TAIs with water leakage during the procedure (50%) was relatively high. However, a similar value was reported by Christensen et al. in their comparison of TAI with conservative bowel management [18]. When considering the 56 TAIs, stools were eliminated in 47 procedures (83.9%). By definition, this percentage was 100% for “relevant” TAIs. This encourages us to think that if our restriction to relevant TAIs is a source of bias, it is a relatively minor bias.

With regard to safety, we did not observe any SAEs. The AEs that did occur (abdominal pain, and mild rectal bleeding in a patient with hemorrhoids at baseline) were generally mild, transient, are well known in the literature [16] and were probably or certainly related to the TAI procedure rather than to the IryPump® R Set device per se. However, the present study was not a sufficiently powered head-to-head study with a non-inferiority design, and we do not know how the IryPump® R Set compares with the Peristeen® in terms of safety and efficacy.

Familiarity with a device appears to be a key factor: despite high ratings (>70%) for functional aspects of the IryPump®R Set, only 50% of the participants were willing to consider use of this new device in the future. However, the participants had been using Peristeen® satisfactorily for at least 6 weeks (the inclusion criterion), and usually much longer (4.3 ± 2.3 years, on average). Long-term TAI users may become accustomed to their chosen TAI modality and may therefore have trouble switching to a new modality—even if the latter offers potential benefits. Conversely, the inclusion of TAI-naïve participants in a clinical study is also likely to disfavor a new device because problems with the TAI procedure may predominate. In theory, the ideal study design would be a randomized, controlled, head-to-head trial of two or more devices in TAI-naïve participants. However, only one randomized, controlled trial has addressed the safety and efficacy of TAI (with the Peristeen®); in a population of 87 patients with spinal cord injury, TAI was found to provide significant benefits, relative to conservative bowel management alone [18].

### Study limitations and strengths

The present study had some limitations. First, the sample size (*n*=15 enrolled patients) was small for a multicenter study, and is likely to have increased the variability of the results. However, the inclusion criteria were relatively strict, and this sample size reflected the three center’s recruitment capacities and the small size of the target group of patients. Hence, we did not calculate the target sample size on the basis of statistical power calculations. Second, the range of pathologies among the study participants was very broad, with both neurologic and non-neurologic conditions. While this reflected the potential “real-life” use of the device, it also constituted a major source of bias in the results. Third, our study’s multicenter design meant that not all patients were trained in use of the new device by the same staff, constituting a source of variability. However, the investigators in all three centers were introduced to the device by the same study monitor. Fourth, the 1-month data collection period was short, and the maximum number of consecutive TAIs studied per participant (5) was low; however, the length of the study period was limited by the participants’ high degree of satisfaction with their usual TAI system (i.e., Peristeen®) and their desire (at least before participating in the study) to resume use of the Peristeen® afterwards. Fifth, the study’s premature withdrawal rate (28.6%) was high for a mean follow-up time of around 20 days. By way of a comparison, Juul and Christen reported that in their study of 507 patients introduced to TAI, 216 (43%) were still using the technique after 12 months, 174 (34%) had discontinued the treatment for various reasons, and 117 (23%) had not returned the end-of-study questionnaire [24]. Sixth, this was an open-label study that lacked a comparator group (e.g., conservative care alone, or another TAI device). The participants were informed of the unique design and functional features of the IryPump R Set, relative to the Peristeen® device with which they were familiar. However, as mentioned in the Introduction, only one randomized, controlled trial has been performed in this field of care, along with ~100 observational studies [5, 18]. Seventh, we chose to study the new device with patients who were familiar and satisfied with TAI; TAI-naïve patients’ use of the device would probably have differed. Lastly, our findings in a group of patients in France may not be generalizable to other countries or healthcare systems.

The study also had a number of strengths. The patients were already familiar with the TAI technique (with between 1 and 7 years of use), and so were able to give extensive feedback on each TAI and their use of the device. The study’s multicenter design (in the rehabilitation departments of three French university hospitals) means that it might be possible to extrapolate the results to patients with bowel dysfunction more generally—or at least to those being cared for in the French health system.

Perspectives for further research include confirmation of these results in a larger population, such as a European-wide study of efficacy and safety in different healthcare systems. Furthermore, the device’s cost-effectiveness remains to be assessed, although this is also the case for established devices such as the Peristeen® [8].

## Conclusions

In patients familiar with TAI, this procedure was feasible with the new IryPump®R Set medical device incorporating an electric air and water pump. Levels of patient satisfaction were high—especially with regard to comfort of use and a feeling of security during TAI.

## Electronic supplementary material

Below is the link to the electronic supplementary material.

Supplementary file1 (DOCX 825 kb)
