# Supplementary material for: Use of a new transanal irrigation device for bowel disorder management by patients familiar with the irrigation technique: a prospective, interventional, multicenter pilot study
Source: Tech Coloproctol. 2020 Apr 21;24(7):731–40. doi: 10.1007/s10151-020-02212-x (PMC7297826; doi:10.1007/s10151-020-02212-x)
Supplement: Supplementary file 1 — Supplementary file1 (DOCX 825 kb) [file 10151_2020_2212_MOESM1_ESM.docx]

**Electronic Supplementary Material**. Illustrations of the IryPump®R Set (B.Braun Melsungen AG, Melsungen, Germany).

The IryPump® R Set comprises an IryPump® R Station, an IryPump® water container, an IryTube, and a single-use IryCath®.


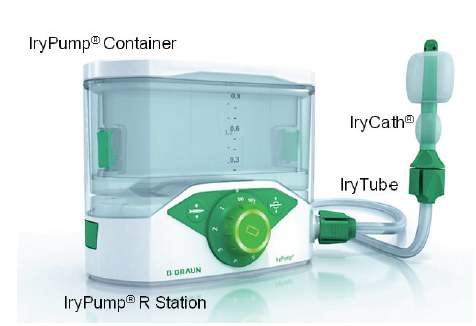


The IryPump® R Station (width: 200 mm - depth: 116 mm - height: 69 mm) consists of a white and green plastic casing, which contains all the electronic and electromechanical elements necessary for its proper functioning. The IryPump® R Station’s components are variously made out of polyphenylene sulphide, ethylene propylene diene monomer rubber, silicone, polyamide 66, polycarbonate, acrylonitrile-butadiene styrene terpolymer, thermoplastic elastomer, stainless steel, and polypropylene. IryPump® R also comprises the IryPump® Container, i.e. a transparent, expandable fluid container with a white, removable lid and temperature indicator (width: 195.6 mm - depth: 87.3 mm, height (collapsed): 110 mm, height (expanded): 182.1 mm (with lid)). The IryPump® Container is made out of polycarbonate, thermoplastic elastomer, metal and silicone.


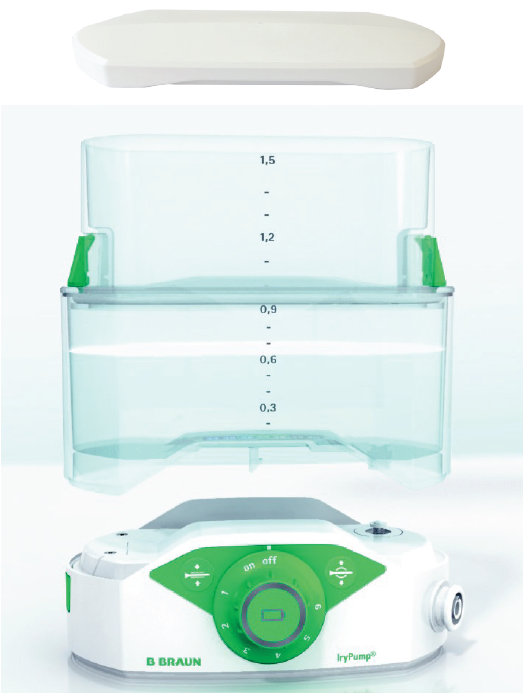


The IryTube tubing connects the IryCath® to the IryPump® R Station. The tubing is made of polyurethane, and the connector are made of thermoplastic urethane containing a green colouring agent. It should be noted that the IryTube® is free of polyvinyl chloride, diethylhexyl phthalate, medicinal substances, radioactive substances, animal tissues and derivatives thereof. The IryTube (with both connectors) is 1340 mm in length, with an outer diameter of 9.9 mm and an inner diameter (a lumen) of 5.3 mm. The air which inflates the double compartment balloon of IryCath® is conducted through a very narrow conduit which runs inside the wall of IryTube from IryPump® R to IryCath® (diameter: 1.2 mm).

The IryCath® is a soft, CH36 rectal catheter with a rounded, open-ended tip and a double-compartment inflatable balloon. The catheter comprises not only, in its centre, a conduit with a large diameter for the irrigation fluid, but also, in its wall, four very narrow conduits for the inflation air of the balloon. The IryCath®’s shaft is made out of thermoplastic elastomer with green colouring agent, and the balloon is made out of thermoplastic elastomer. The IryCath® is coated with lubricant containing glycerol, water, Crovol A70 vegetable oil, and hydroxyethyl cellulose. The total length of the catheter is 112.3 mm. The diameter of the catheter at the tip is 12.2 mm. The length of the portion carrying the proximal (anal) balloon compartment is 29.5 mm. The length of the portion carrying the distal (rectal) balloon compartment is 36.0 mm.
